# Association of IL‐17 and IL‐27 polymorphisms with susceptibility to recurrent pregnancy loss and pre‐eclampsia: A systematic review and meta‐analysis

**DOI:** 10.1002/iid3.1057

**Published:** 2023-10-25

**Authors:** Yue Ma, Mingyue Ma, Shenglong Ye, Yuanying Liu, Xueqing Zhao, Yongqing Wang

**Affiliations:** ^1^ Department of Obstetrics and Gynecology, Peking University Third Hospital National Clinical Research Center for Obstetrical and Gynecology Beijing China; ^2^ Department of Public Health Johns Hopkins University Baltimore Maryland USA

**Keywords:** IL‐17, IL‐27, polymorphism, pre‐eclampsia, recurrent pregnancy loss

## Abstract

**Objective:**

Recurrent pregnancy loss (RPL) and pre‐eclampsia (PE) are immune‐related pregnancy complications that have been linked to CD4^+^ T cells and their cytokines, which can be influenced by genetic polymorphisms. This meta‐analysis aimed to investigate the relationship between interleukin (IL)‐17 and ‐27 polymorphisms and the susceptibility to RPL and PE.

**Methods:**

All eligible case‐control studies published up to February 2023 were identified by searching PubMed, EMBASE, Cochrane, Web of Science, and Google Scholar. The risk of recurrent pregnancy loss and PE associated with the IL‐17 rs2275913, IL‐17 rs763780, IL‐27 rs153109, and IL‐27 rs17855750 polymorphisms were estimated for each study.

**Results:**

The meta‐analysis incorporated a total of 13 studies. The overall analysis indicated that IL‐17 rs2275913, IL‐17 rs763780, IL‐27 rs153109, and IL‐27 rs17855750 polymorphisms were not significantly associated with immune‐related pregnancy complications, including RPL and PE. However, when the analysis was stratified by disease type, the IL‐17 rs2275913 polymorphism was found to be associated with an increased risk of RPL (recessive model AA/GA + GG: OR = 1.68, 95% confidence interval [CI]: 1.13–2.49, *p* = .01).

**Conclusions:**

The IL‐17 rs763780, IL‐27 rs153109, and IL‐27 rs17855750 polymorphisms were not significantly associated with RPL and PE, whereas the IL‐17 rs2275913 polymorphism was associated with the susceptibility to recurrent miscarriage.

## Graphical abstract

Pre‐eclampsia and recurrent pregnancy loss are both classified as immune‐related pregnancy complications due to their similar immunological pathogenesis. The interleukin (IL)‐17 rs763780, IL‐27 rs153109, and IL‐27 rs17855750 polymorphisms were not significantly associated with pre‐eclampsia and recurrent pregnancy loss. The IL‐17 rs2275913 polymorphism was associated with the susceptibility to recurrent pregnancy loss.

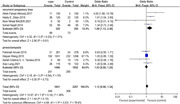

## 1 INTRODUCTION

A successful pregnancy hinges on achieving immune balance within the mother, a process that involves various immune cells. When this delicate equilibrium falters due to immune dysregulation, it can lead to adverse pregnancy outcomes. Among these immune cells, T cells assume a pivotal role in the maternal immune system. During pregnancy, they modulate maternal‐fetal immune homeostasis by releasing a variety of cytokines. Previous investigations have unearthed an imbalance in CD4^+^ T cells and their associated cytokines in immune‐related pregnancy complications like recurrent pregnancy loss (RPL) and pre‐eclampsia (PE).^1^, ^2^, ^3^

Interleukin (IL)‐17 and IL‐27 are noteworthy cytokines intricately connected with CD4^+^ T cells. IL‐17, the primary effector cytokine of T helper 17 (Th17) cells, notably instigates inflammatory responses. On the other hand, IL‐27 exerts an influence on CD4^+^ T cell differentiation. It prompts naïve T cells to differentiate into T helper 1 (Th1) cells while impeding their differentiation into T helper 2 (Th2) and Th17 cells.^4^, ^5^, ^6^ Notably, research has unveiled a significant elevation in the levels of IL‐17 and ‐27 in patients grappling with immune‐related pregnancy complications in comparison to women experiencing uncomplicated pregnancies.^7^, ^8^, ^9^ These findings underscore the potentially pivotal role of IL‐17 and ‐27 in the pathogenesis and progression of immune‐related pregnancy complications.

Single nucleotide polymorphisms (SNPs), a common type of heritable variation within the human genome, possess the capacity to influence protein activity or expression by modifying gene function or transcriptional activity. SNPs situated within certain cytokine genes can to modulate cytokine production, thereby resulting in disparate cytokine levels. Consequently, they are associated with a spectrum of diseases. Notably, IL‐17 rs2275913 and IL‐17 rs763780 are SNPs intricately linked with IL‐17 secretion. Similarly, rs153109 and rs17855750 represent the most prevalent polymorphisms within the IL‐27 gene loci and have been associated with the development of diverse conditions, including autoimmune diseases. Despite numerous studies delving into the correlation between these polymorphisms—IL‐17 rs2275913, rs763780, and IL‐27 rs153109, rs17855750—and immune‐related pregnancy complications, the findings have exhibited significant variability. As a result, we conducted a comprehensive meta‐analysis to investigate the potential associations between immune‐related pregnancy complications and the IL‐17 rs2275913, IL‐17 rs763780, IL‐27 rs153109, and IL‐27 rs17855750 polymorphisms.

## 2 MATERIALS AND METHODS

The meta‐analysis adhered to the PRISMA guidelines and its protocol was registered with INPLASY (protocol ID: INPLASY2022120007).

### 2.1 Literature search strategy

Online databases such as PubMed, EMBASE, Cochrane, Web of Science, and Google Scholar were searched for relevant literature up to February 2023, with the following keywords “IL‐17,” “IL‐27,” “rs2275913,” “rs763780,” “rs153109,” “rs17855750,” “polymorphism,” “pre‐eclampsia,” and “recurrent pregnancy loss.”

### 2.2 Data extraction and quality evaluation

Two authors independently searched and screened the literature in accordance with the inclusion and exclusion criteria, extracted data, and assessed study quality. Any disagreement between the two authors was resolved by consensus between the authors. If no agreement was reached, a third author was consulted. The extracted data included the first author of the literature, the year of publication, the type of literature, country, the sample sizes of the patient and control groups, the frequency of a genotype, and the method for genotype detection. PE was defined as elevated blood pressure (systolic blood pressure ≥140 mmHg and/or diastolic blood pressure ≥90 mmHg) occurring after 20 weeks of gestation accompanied by positive urine protein or 24 h proteinuria exceeding 0.3 g. It may be accompanied by other severe reactions, such as headache, dizziness, nausea and vomiting, epigastric pain, impaired liver and kidney function, thrombocytopenia, coagulation dysfunction, and intrauterine fetal developmental delay. RPL was defined as the occurrence of two or more consecutive spontaneous abortions. The Newcastle‐Ottawa Scale was utilized to evaluate the quality of the literature.

### 2.3 Inclusion and exclusion criteria

Inclusion criteria: (1) The studies focused on the association of IL‐17 and ‐27 polymorphisms with RPL and PE. (2) All studies were designed as case‐control studies, with cases being defined as patients with either PE or RPL, but with exclusion of miscarriages due to known causes such as autoimmune diseases, reproductive tract abnormalities, or chromosomal abnormalities. The control group was comprised of normal pregnant women. (3) The studies had sufficient data, and odds ratios (OR) and 95% confidence intervals (CI) were calculated.

Exclusion criteria: (1) Letters, reviews, and single case reports. (2) Duplicate published literature. (3) Irrelevant to the association between these genetic variations and immune‐related pregnancy complications. (4) Literature with incomplete data or unavailable genotype frequencies or allele frequencies. (5) Nonhuman experiments. (6) Unable to access complete research articles. (7) Not designed as a case‐control study.

### 2.4 Statistical analysis

RevMan software, version 5.4.1, and Stata software, version 15.1, were employed to perform a meta‐analysis of the data. The Hardy–Weinberg equilibrium (HWE) test was performed on the genotypes of the control group included in the study, with *p* < .05 indicating noncompliance with HWE. The included literature was tested for heterogeneity (*Q* and *I* ^2^ tests), and if *p* < 0.05 or *I* ^2^ > 50%, a random‐effects model was selected to combine the data for analysis, and a fixed‐effects model was used otherwise. Significant heterogeneity was addressed by using subgroup, sensitivity, or descriptive analysis. ORs with 95% CIs were applied as the effect size for the evaluation of the associations of IL‐17 and ‐27 polymorphisms with RPL and PE susceptibility. The following models were utilized to evaluate the associations of IL‐17 and ‐27 polymorphisms with the risk of recurrent spontaneous abortion (RPL) and PE: allelic, dominant, recessive, heterozygous, and homozygote models. To examine the potential impact of publication bias on the accuracy of the results, we created funnel plots. Additionally, we utilized Egger's linear regression test to evaluate the symmetry of the funnel plot. Two‐sided *p* < .05 was considered statistically significant.

## 3 RESULTS

### 3.1 Study selection and characteristics

**Figure 1:**
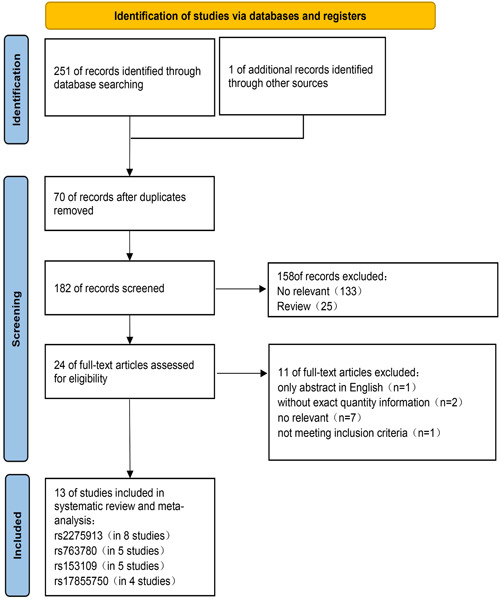
Flowchart of literature search and selection process.

The literature search process is shown in Figure 1. The initial literature search retrieved 252 relevant studies. After the titles and abstracts were read, 158 articles were excluded because of apparent irrelevance or duplication or because they were review articles. Twenty‐four studies were selected for full‐text assessment, of which 11 were excluded due to missing data, non‐English language, not meeting inclusion criteria and nonrelevance. Finally, the meta‐analysis incorporated a total of 13 studies.^10^, ^11^, ^12^, ^13^, ^14^, ^15^, ^16^, ^17^, ^18^, ^19^, ^20^, ^21^, ^22^ The specific characteristics of each included study are shown in Table 1.

**Table 1:** The demographic and clinical characteristics of cases and controls.

Polymorphism	First author	Year	Country	Pregnancy complication	Sample size (case/control)	Method	Case			Control			Hardy‐Weinberg equilibrium (*p*)	The Newcastle‐Ottawa scale
							AA	AB	BB	AA	AB	BB		
IL‐17 rs2275913	Afrah Fahad Alkhuriji	2017	Saudi Arabia	Recurrent pregnancy loss	100/100	Taqman probe method	56	33	11	67	30	3	.871	8
	Fahimah Anvari	2015	Iran	Pre‐eclampsia	261/278	PCR‐RFLP/Q‐PCR	67	173	21	66	188	24	<.001	7
	Haidy E. Zidan	2015	Egyp	Recurrent pregnancy loss	120/120	PCR‐RFLP	34	50	36	49	49	22	.126	8
	Haiyan Wang	2015	China	Pre‐eclampsia	1031/1298	RT‐PCR	366	473	192	466	600	232	.109	8
	Noor Nihad Baper	2021	Iraq	Recurrent abortion	50/50	RT‐PCR	33	17	0	28	20	2	.495	5
	Sarah Cristina S. V. Tanaka	2019	Brazil	Pre‐eclampsia	89/174	RT‐PCR	59	28	2	99	65	10	.876	8
	Soheil Najafi	2014	Iran	Recurrent pregnancy loss	85/85	PCR‐RFLP	7	26	52	3	36	46	.210	7
	Xiao Lang	2021	China	Pre‐eclampsia	115/102	PCR‐RFLP or TaqMan probe method	24	65	26	39	44	19	.299	8
IL‐17 rs763780	Afrah Fahad Alkhuriji	2017	Saudi Arabia	Recurrent pregnancy loss	100/100	Taqman probe method	5	84	11	90	9	1	.180	8
	Fahimah Anvari	2015	Iran	Pre‐eclampsia	261/278	PCR‐RFLP/Q‐PCR	216	36	9	242	31	5	.002	7
	Haidy E. Zidan	2015	Egyp	Recurrent pregnancy loss	120/120	PCR‐RFLP	53	50	17	37	51	32	.104	8
	Haiyan Wang	2015	China	Pre‐eclampsia	1031/1298	RT‐PCR	838	185	8	1036	245	17	.561	8
	Soheil Najafi	2014	Iran	Recurrent pregnancy loss	85/85	PCR‐RFLP	37	42	6	22	60	3	<.001	7
IL‐27 rs153109	Azam Aramesh	2021	Iran	Pre‐eclampsia	199/228	PCR‐RFLP	82	84	33	82	117	29	.200	8
	Bin Liu	2016	China	Pre‐eclampsia	1040/1247	Taqman probe method	405	431	198	467	608	165	.135	8
	Danial Jahantigh	2020	Iran	Pre‐eclampsia	170/170	PCR‐RFLP	73	81	16	96	67	7	.263	9
	Peng Chen	2016	China	Pre‐eclampsia	212/451	PCR‐RFLP	96	92	24	162	210	79	.443	8
	Zeinab Nematollahi	2015	Iran	Recurrent pregnancy loss	150/150	PCR‐RFLP	60	74	16	73	65	12	.638	8
IL‐27 rs17855750	Azam Aramesh	2021	Iran	Pre‐eclampsia	199/228	PCR‐RFLP	99	59	41	106	80	42	<.001	8
	Bin Liu	2016	China	Pre‐eclampsia	1040/1247	Taqman probe method	763	224	27	885	291	34	.093	8
	Danial Jahantigh	2020	Iran	Pre‐eclampsia	170/170	PCR‐RFLP	62	89	19	79	84	7	.008	9
	Peng Chen	2016	China	Pre‐eclampsia	212/451	PCR‐RFLP	166	42	4	364	78	9	.054	8

### 3.2 Quantitative data synthesis

#### 3.2.1 IL‐17 rs2275913 polymorphism

Overall, quantitative analysis revealed no statistically significant association between IL‐17 rs2275913 polymorphism and immune‐related pregnancy complications in all genetic models: allelic model (G/A): OR = 1.12, 95% CI: 0.91–1.36, *p* = .29; dominant model (AA + GA/GG): OR = 1.01, 95% CI: 0.77–1.33, *p* = .92; recessive model (AA/GA + GG): OR = 1.13, 95% CI: 0.96–1.34, *p* = .15; heterozygous model (GA/GG): OR = 1.05，95% CI: 0.81–1.37, *p* = .69; homozygote model (AA/GG): OR = 1.24, 95% CI: 0.77–1.99, *p* = .38.

**Figure 2:**
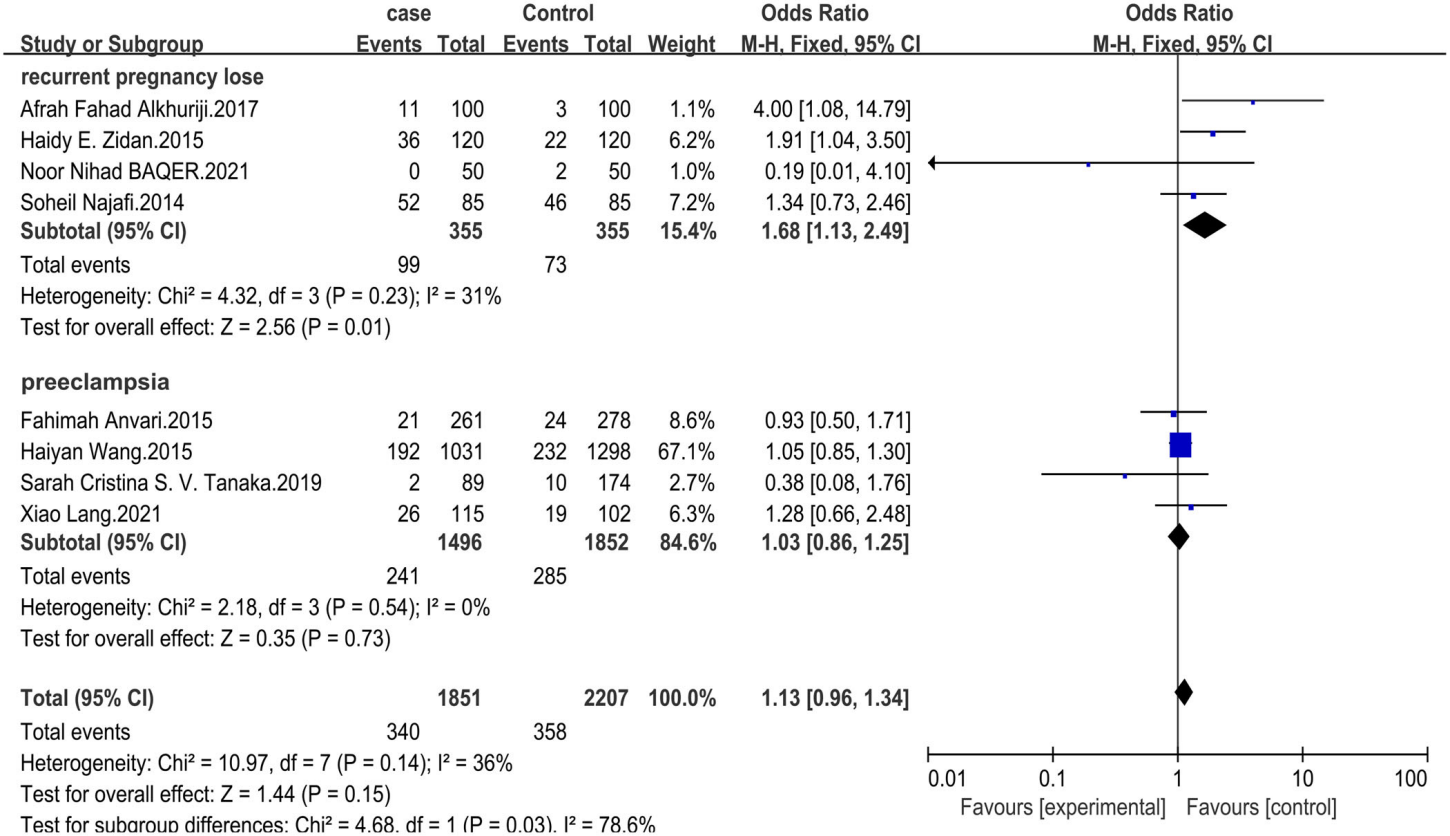
The forest plot for IL‐17 rs2275913 in recurrent pregnancy loss (RPL) and Pre‐eclampsia (PE) in the recessive model.

Furthermore, stratified analysis by disease type revealed that IL‐17 rs2275913 was significantly associated with the risk of RPL in the recessive model ([AA/GA + GG]: OR = 1.68, 95% CI: 1.13–2.49, *p* = .01 [Figure 2]), but not in other genetic models (allelic model [G/A]: OR = 1.26, 95% CI: 0.86–1.85, *p* = .23; dominant model [AA + GA/GG]: OR = 1.16, 95% CI: 0.62–2.17, *p* = .64; heterozygous model [GA/GG]: OR = 1.10, 95% CI: 0.77–1.58, *p* = .60; homozygote model [AA/GG]: OR = 1.43, 95% CI: 0.47–4.32, *p* = .53). Furthermore, IL‐17 rs763780 had no statistically significant association with PE in any of the genetic models: allelic model (G/A): OR = 1.02, 95% CI: 0.82–1.26, *p* = .86; dominant model (AA + GA/GG): OR = 0.97, 95% CI: 0.83–1.12, *p* = .66; recessive model (AA/GA + GG): OR = 1.03, 95% CI: 0.86–1.25, *p* = .73; heterozygous model (GA/GG): OR = 1.06, 95% CI: 0.75–1.50, *p* = .73; homozygote model (AA/GG): OR = 1.06, 95% CI: 0.86–1.31, *p* = .57 (Table 2).

**Table 2:** Summary of meta‐analysis for the association between interleukin (IL)‐17 rs2275913 polymorphism and risk of immune‐related pregnancy complications.

	Genetic model	Type of model	Heterogeneity		Odds ratio (OR)			
			*I* ^2^ (%)	*P*_H_	OR	95% confidence interval (CI)	*Z*_test_	*P*_OR_
Overall	G/A	Random	65	0.006	1.12	0.91–1.36	1.06	0.29
	AA + GA/GG	Random	52	0.05	1.01	0.77–1.33	0.10	0.92
	AA/GA + GG	Fixed	36	0.14	1.13	0.96–1.34	1.44	0.15
	GA/GG	Random	51	0.05	1.05	0.81–1.37	0.40	0.69
	AA/GG	Random	60	0.01	1.24	0.77–1.99	0.08	0.38
Disease type								
Recurrent pregnancy loss	G/A	Random	59	0.06	1.26	0.86–1.85	1.21	0.23
	AA + GA/GG	Random	59	0.06	1.16	0.62–2.17	0.47	0.64
	AA/GA + GG	Fixed	31	0.23	1.68	1.13–2.49	2.56	0.01
	GA/GG	Fixed	43	0.15	1.10	0.77–1.58	0.53	0.60
	AA/GG	Random	62	0.05	1.43	0.47–4.32	0.63	0.53
Pre‐eclampsia	G/A	Random	62	0.05	1.02	0.82–1.26	0.17	0.86
	AA + GA/GG	Fixed	12	0.32	0.97	0.83–1.12	0.45	0.66
	AA/GA + GG	Fixed	0	0.54	1.03	0.86–1.25	0.35	0.73
	GA/GG	Random	66	0.03	1.06	0.75–1.50	0.34	0.73
	AA/GG	Fixed	49	0.11	1.06	0.86–1.31	0.56	0.57

#### 3.2.2 IL‐17 rs763780 polymorphism

Overall, quantitative analysis showed that IL‐17 rs763780 had no significant association with immune‐related pregnancy complications in all genetic models: allelic model (C/T): OR = 1.54, 95% CI: 0.69–3.41, *p* = .29; dominant model (CC + TC/TT): OR = 1.96, 95% CI: 0.67–5.77, *p* = .22; recessive model (CC/TC + TT): OR = 1.27, 95% CI: 0.50–3.19, *p* = .62; heterozygous model (TC/TT): OR = 1.98, 95% CI: 0.66–5.93, *p* = .22; homozygote model (CC/TT): OR = 1.88, 95% CI: 0.46–7.70, *p* = .38. Stratified analysis by disease type revealed no significant association between IL‐17 rs763780 and RPL (allelic model [C/T]: OR = 1.98, 95% CI: 0.32–12.45, *p* = .46; dominant model [CC + TC/TT]: OR = 3.37, 95% CI: 0.18–63.09, *p* = .42; recessive model [CC/TC + TT]: OR = 1.85, 95% CI: 0.29–11.82, *p* = .51; heterozygous model [TC/TT]: OR = 3.47, 95% CI: 0.19–65.22, *p* = .41; homozygote model [CC/TT]: OR = 3.76, 95% CI: 0.17–83.65, *p* = .40) and between IL‐17 rs763780 and PE (allelic model [C/T]: OR = 1.10, 95% CI: 0.69–1.74, *p* = .70; dominant model [CC + TC/TT]: OR = 1.07, 95% CI: 0.71–1.61, *p* = .75; recessive model [CC/TC + TT]: OR = 1.01, 95% CI: 0.32–3.26, *p* = .98; heterozygous model [TC/TT]: OR = 1.01, 95% CI: 0.77–1.34, *p* = .94; homozygote model [CC/TT]: OR = 1.03, 95% CI: 0.30‐3.45, *p* = .97 [Table 3]).

**Table 3:** Summary of meta‐analysis for the association between interleukin (IL)‐17 rs763780 polymorphism and risk of immune‐related pregnancy complications.

Subgroup	Genetic model	Type of model	Heterogeneity		Odds ratio (OR)			
			*I* ^2^ (%)	*P*_H_	OR	95% confidence interval (CI)	*Z*_test_	*P*_OR_
Overall	C/T	Random	96	<0.001	1.54	0.69–3.41	1.06	0.29
	CC + TC/TT	Random	96	<0.001	1.96	0.67–5.77	1.23	0.22
	CC/TC + TT	Random	73	0.005	1.27	0.50–3.19	0.50	0.62
	TC/TT	Random	96	<0.001	1.98	0.66–5.93	1.23	0.22
	CC/TT	Random	88	<0.001	1.88	0.46–7.70	0.87	0.38
Disease type								
Recurrent pregnancy loss	C/T	Random	98	<0.001	1.98	0.32–12.45	0.73	0.46
	CC + TC/TT	Random	98	<0.001	3.37	0.18–63.09	0.81	0.42
	CC/TC + TT	Random	83	0.003	1.85	0.29–11.82	0.65	0.51
	TC/TT	Random	98	<0.001	3.47	0.19–65.22	0.83	0.41
	CC/TT	Random	93	<0.001	3.76	0.17–83.65	0.84	0.40
Pre‐eclampsia	C/T	Random	75	0.04	1.10	0.69–1.74	0.39	0.70
	CC + TC/TT	Random	62	0.1	1.07	0.71–1.61	0.32	0.75
	CC/TC + TT	Random	65	0.09	1.01	0.32–3.26	0.02	0.98
	TC/TT	Random	27	0.24	1.01	0.77–1.34	0.08	0.94
	CC/TT	Random	67	0.08	1.03	0.30–3.45	0.04	0.97

#### 3.2.3 IL‐27 rs153109 polymorphism

IL‐27 rs153109 showed no statistically significant association with immune‐related pregnancy complications in all genetic models: allelic model (G/A): OR = 1.07, 95% CI: 0.85–1.35, *p* = .55; dominant model (GG + AG/AA): OR = 1.02, 95% CI: 0.76–1.36, *p* = .92; recessive model (GG/AG + AA): OR = 1.27, 95% CI: 0.83–1.95, *p* = .27; heterozygous model (AG/AA): OR = 0.96, 95% CI: 0.72–1.27, *p* = .76; homozygote model (GG/AA): OR = 1.22, 95% CI: 0.75–2.00, *p* = .42. Stratified analysis by disease type revealed no significant association between IL‐27 rs153109 and RPL (allelic model [G/A]: OR = 1.30, 95% CI: 0.92–1.86, *p* = .14; dominant model [GG + AG/AA]: OR = 1.42, 95% CI: 0.90–2.25, *p* = .13; recessive model [GG/AG + AA]: OR = 1.37, 95% CI: 0.63–3.01, *p* = .43; heterozygous model [AG/AA]: OR = 1.39, 95% CI: 0.865–2.23, *p* = .18; homozygote model [GG/AA]: OR = 1.62, 95% CI: 0.71–3.69, *p* = .25) and between IL‐27 rs153109 and PE (allelic model [G/A]: OR = 1.03, 95% CI: 0.79–1.35, *p* = .80; dominant model [GG + AG/AA]: OR = 1.95, 95% CI: 0.69–1.30, *p* = .74; recessive model [GG/AG + AA]: OR = 1.26, 95% CI: 0.75–2.10, *p* = .38; heterozygous model [AG/AA]: OR = 0.89, 95% CI: 0.66–1.18, *p* = .42; homozygote model [GG/AA]: OR = 1.17, 95% CI: 0.65–2.08, *p* = .60 [Table 4]).

**Table 4:** Summary of meta‐analysis for the association between interleukin (IL)‐27 rs153109 polymorphism and risk of immune‐related pregnancy complications.

Subgroup	Genetic model	Type of model	Heterogeneity		Odds ratio (OR)			
			*I* ^2^ (%)	*P*_H_	OR	95% confidence interval (CI)	Z_test_	*P*_OR_
Overall	G/A	Random	77	0.001	1.07	0.85–1.35	0.61	0.55
	GG + AG/AA	Random	74	0.004	1.02	0.76–1.36	0.10	0.92
	GG/AG + AA	Random	70	0.009	1.27	0.83–1.95	1.09	0.27
	AG/AA	Random	69	0.01	0.96	0.72–1.27	0.31	0.76
	GG/AA	Random	74	0.003	1.22	0.75–2.00	0.80	0.42
Disease type								
Recurrent pregnancy loss	G/A	Random	/	/	1.30	0.92–1.82	1.48	0.14
	GG + AG/AA	Random	/	/	1.42	0.90–2.25	1.51	0.13
	GG/AG + AA	Random	/	/	1.37	0.63–3.01	0.79	0.43
	AG/AA	Random	/	/	1.39	0.86–2.23	1.34	0.18
	GG/AA	Random	/	/	1.62	0.71–3.69	1.15	0.25
Pre‐eclampsia	G/A	Random	82	0.001	1.03	0.79–1.35	0.25	0.80
	GG + AG/AA	Random	75	0.007	0.95	0.69–1.30	0.33	0.74
	GG/AG + AA	Random	78	0.004	1.26	0.75–2.10	0.88	0.38
	AG/AA	Random	67	0.03	0.89	0.66–1.18	0.81	0.42
	GG/AA	Random	8	0.02	1.17	0.65–2.08	0.52	0.60

#### 3.2.4 IL‐27 rs17855750 polymorphism

This meta‐analysis observed no significant association between IL‐27 rs17855750 and the risk of PE in all genetic models: allelic model (G/T): OR = 1.07, 95% CI: 0.87–1.32, *p* = .52; dominant model (GG + GT/TT): OR = 1.04, 95% CI: 0.82–1.31, *p* = .76; recessive model (GG/GT + TT): OR = 1.24, 95% CI: 0.80–1.91, *p* = .34); heterozygous model (GT/TT): OR = 0.96, 95% CI: 0.82–1.12, *p* = .61; homozygote model (GG/AA): OR = 1.25, 95% CI: 0.74–2.09, *p* = .40 (Table 5).

**Table 5:** Summary of meta‐analysis for the association between interleukin (IL)‐27 rs17855750 polymorphism and risk of pre‐eclampsia.

Pre‐eclampsia	Genetic model	Type of model	Heterogeneity		Odds ratio (OR)			
			*I* ^2^ (%)	*P*_H_	OR	95% confidence interval (CI)	Z_test_	*P*_OR_
Overall	G/T	Random	57	0.07	1.07	0.87–1.32	0.64	0.52
	GG + GT/TT	Fixed	47	0.13	0.98	0.85–1.14	0.23	0.82
	GG/GT + TT	Fixed	38	1.18	1.20	0.88–1.64	1.16	0.25
	GT/TT	Fixed	33	0.22	0.96	0.82–1.12	0.51	0.61
	GG/AA	Random	53	0.10	1.25	0.74–2.09	0.84	0.40

### 3.3 Publication bias and sensitivity analysis

Sensitivity analysis was implemented in the studies focusing on immune‐related pregnancy complications. Individual studies were sequentially excluded to evaluate the influence of each study on the overall pooled ORs. According to the findings, none of the individual studies had a noteworthy impact on the pooled ORs. Moreover, sensitivity analysis was carried out after studies departing from the HWE were excluded. The results were not changed in the overall population, indicating that the results were statistically reliable and robust.

**Figure 3:**
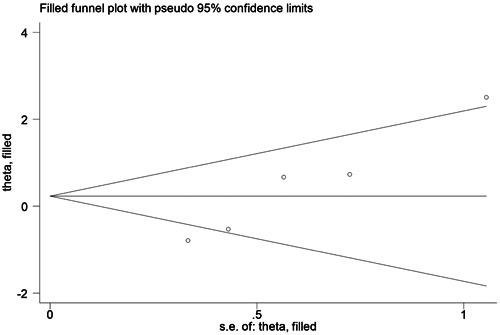
Funnel plot after adjusted by the trim‐and‐fill method for the association between interleukin (IL)‐17 rs763780 CC/TC polymorphism and risk of immune‐related pregnancy complications.

**Figure 4:**
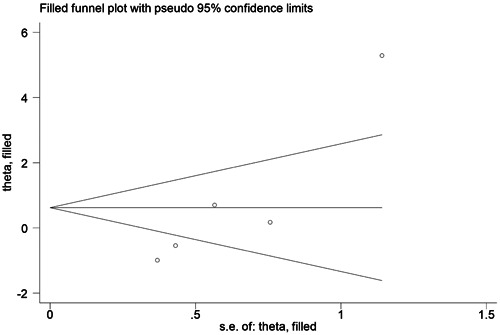
Funnel plot after adjusted by the trim‐and‐fill method for the association between interleukin (IL)‐17 rs763780 CC/TT polymorphism and risk of immune‐related pregnancy complications.

Significant publication bias for IL‐17 rs763780 CC/TC + TT and CC/TT genotypes was detected by Egger's test (*p* = .006, *p* = .028, respectively). We confirmed the presence of publication bias using the trim‐and‐fill method, and the results remained unchanged, indicating the robustness of our findings (IL‐17 rs763780 CC/TC: OR = 1.260, 95% CI = 0.505‐3.142, and IL‐17 rs763780 CC/TT: OR = 1.862, 95% CI = 0.464‐7.471) (Figures 3 and 4).

## 4 DISCUSSION

Maintaining immune tolerance and the delicate balance of the maternal‐fetal immune microenvironment is crucial for a successful pregnancy. CD4^+^ T cells, which are responsible for the immune response, have a significant role in this process. CD4^+^ T cells consist of T helper (Th1, Th2, Th9, Th17, Th22, and T follicular helper cells) and regulatory T cells (Treg) that produce various cytokines.^23^ Cytokines affect multiple aspects of pregnancy by controlling embryonic implantation, cell growth, angiogenesis, invasion of trophoblast cells, spiral artery remodeling, and maintaining maternal‐fetal interface tolerance.^24^ Evidence shows that pregnancy complications, such as miscarriage, preterm delivery, and PE, are closely linked to the balance of Th1/Th2 and Th17/Treg cells and their cytokines.^2^, ^25^

PE and RPL are considered to have similar immunological pathogenesis and are classified as immune‐related pregnancy complications by some studies.^1^ Previous studies have shown that women with RPL and PE have a higher proportion of Th17 cells, increased levels of IL‐17, and decreased proportions of Treg cells in their peripheral blood and decidual tissue when compared to women with normal pregnancies.^26^, ^27^, ^28^, ^29^ IL‐17 is the main cytokine of Th17 cells, which binds to its receptor IL‐17R and triggers downstream signaling pathways to stimulate the release of inflammatory molecules, such as granulocyte colony‐stimulating factor, and chemokines CXCL1 and CXCL2, IL‐6, IL‐8, resulting in the recruitment and activation of neutrophils, the generation of reactive oxygen species, inflammatory responses, tissue damage, and an increased risk of RPL and PE.^30^, ^31^ In a mouse model, administering IL‐17 via intraperitoneal injection during pregnancy resulted in abortion, while treating susceptible mice with anti‐IL‐17 antibodies decreased abortion rates.^2^ In a rat model of PE with reduced uteroplacental perfusion (RUPP), Th17 cell levels and IL‐17 were elevated. Treatment with soluble IL‐17 receptor C in RUPP rats, which blocked the IL‐17 signaling cascade, led to a notable reduction in the count of Th17 cells in peripheral blood and a marked reduction in blood pressure.^32^, ^33^

The production of cytokines can be altered by genetic polymorphisms. Two of the most extensively studied SNPs of IL‐17 are rs2275913 and rs763780. Rs2275913 is situated in a binding motif for nuclear factor‐activated T cells and is essential for controlling IL‐17 transcription. It demonstrates robust promoter activity, ultimately governing the expression of IL‐17. The presence of rs2275913 has been suggested to increase the secretion of IL‐17.^34^ Besides, the polymorphic gene rs763780 of IL‐17F can also affect the expression of IL‐17. Numerous studies have explored the correlation between IL‐17 polymorphisms and RPL as well as PE, but their findings are conflicting. While some studies found no significant correlation between IL‐17 polymorphisms and the risk of RPL or PE, others reported an association between certain genotypes and increased or decreased risk of these conditions. The inconsistencies in the conclusions may be due to various factors such as small sample sizes, ethnic differences, and differences in statistical methods. This meta‐analysis utilized different genetic models such as allelic, dominant, recessive, heterozygote, and homozygote models to assess the association of IL‐17 gene polymorphisms with RPL or PE, two immune‐related pregnancy complications with high prevalence. The results suggested that while the five genetic models of rs2275913 did not exhibit any significant differences between the case and control cohorts, there was a statistically significant variance in the distribution of homozygous mutant type AA between the RPL and control cohorts. This finding suggests that the mutant genotype AA of the IL‐17 rs2275913 polymorphism is significantly associated with the occurrence of recurrent miscarriage. Hence, delving into the etiology of RPL through the IL‐17 polymorphic locus promises to deepen our comprehension of RPL's pathogenesis. This, in turn, can establish a robust foundation for clinical prevention and treatment while also potentially opening up a novel genetic‐level approach to diagnosing and managing RPL. Such advancements hold the potential to enhance pregnancy outcomes and reduce the occurrence of pregnancy‐related complications.

IL‐27 is a cytokine with pleiotropic effects that has been demonstrated to have a dual function in modulating inflammation. On one hand, IL‐27 can promote the differentiation of Th1 cells and promote the production of pro‐inflammatory cytokines like IFN‐γ, while also exerting pro‐inflammatory effects by inhibiting the differentiation and function of Treg cells.^5^ On the other hand, IL‐27 can suppress the expression and secretion of IL‐17, while promoting the secretion of IL‐10, an anti‐inflammatory cytokine, thereby suppressing the inflammatory response.^4^ Previous studies have reported reduced IL‐27 expression in the decidual tissue of RPL patients.^35^ However, the role of IL‐27 in PE remains controversial. While Jahantigh found a positive correlation between elevated IL‐27 levels and PE severity,^9^ Yin's research showed no notable contrast in serum IL‐27 levels between the control and PE groups but noticed a surge in the expression of IL‐27 and its receptors in trophoblast cells in PE.^36^ Additionally, Zhang found that IL‐27 can encourage the proliferation, migration, and invasion of trophoblast cells,^37^ while Ge found that IL‐27 regulates the epithelial‐mesenchymal transition of trophoblast cells, thereby inhibiting their migratory and invasive capacity, and contributing to the pathogenesis of PE.^38^ These discrepancies could be due to differences in sample size, time of onset of PE, and IL‐27 concentrations applied in in vitro experiments. The role of IL‐27 as a pleiotropic cytokine in pregnancy complications requires further exploration. The main polymorphic loci studied so far include rs153109 and rs17855750, with inconsistent findings on their association with PE and RPL. Our meta‐analysis did not find an association between IL‐27 rs153109 and rs17855750 polymorphisms and either disease in the five genetic models studied. However, since we only incorporated a limited number of studies, more comprehensive and well‐designed research is necessary in the future to verify the outcomes of this meta‐analysis.

Nevertheless, this meta‐analysis has several limitations. First, only articles published in English were included, which increases the risk of language bias. Second, despite subgroup analysis, significant heterogeneity was observed among studies, potentially due to variations in ethnicity, region, sample size, and assay method. The considerable heterogeneity may have affected the reliability of the results to some extent. Third, publication bias was present in the results of IL‐17 rs763780 CC/TC + TT and CC/TT polymorphisms, although the stability of the findings was confirmed by the trim‐and‐fill method. Fourth, there is a possibility that some relevant literature was missed during the literature search, and certain articles were excluded because individual genotype data were unavailable. Finally, RPL and PE are complex diseases with multiple factors, genes, and mechanisms involved, and the potential interactions between genes and the environment were not explored in this study.

In recent times, the prevalence of RPL and PE has been on the rise. These conditions are complex diseases influenced by various factors such as genetic defects, immunological abnormalities, and environmental factors. Immunological and genetic factors have received increasing attention in the study of RPL and PE. Multiple gene mutations have been identified as potential contributors to the development of these diseases, and the accumulation of these small mutations in causative genes may increase disease susceptibility. Therefore, identifying closely associated genes is crucial. In this study, we conducted a meta‐analysis to explore the link between IL‐17 and ‐27 gene polymorphisms and RPL and PE risk. The findings indicated that IL‐17 rs2275913 polymorphism was linked to susceptibility to RPL, while IL‐17 rs763780, IL‐27 rs153109, and IL‐27 rs17855750 polymorphisms were not significantly associated with immune‐related pregnancy complications. These results offer valuable information for identifying high‐risk groups at risk of RPL and PE, and they provide a molecular biological foundation for the early diagnosis and treatment of immune‐related pregnancy complications. However, as these conditions are caused by multiple genes, this study has limitations and does not reflect the comprehensive relationship between genes and diseases. Therefore, additional investigation is necessary to examine the interaction of different loci among multiple susceptibility genes and their impact on disease susceptibility.

## AUTHOR CONTRIBUTIONS

**Yue Ma**: Conceptualization; data curation; formal analysis; investigation; methodology; software; validation; visualization; writing—original draft; writing—review and editing. **Mingyue Ma**: Data curation; formal analysis; supervision. **Shenglong Ye**: Data curation; formal analysis; software; supervision. **Yuanying Liu**: Data curation; formal analysis; software. **Xueqing Zhao**: Formal analysis; software; supervision. **Yongqing Wang**: Conceptualization; funding acquisition; methodology; resources; supervision; writing—review and editing.

## CONFLICT OF INTEREST STATEMENT

The authors declare no conflict of interest.

## ETHICS STATEMENT

This meta‐analysis did not involve primary data collection or intervention with human participants; therefore, ethics approval was not required.
